# Investigation of subarachnoid haemorrhage: Does the buck stop with CT?

**Published:** 2010-08-25

**Authors:** P Mehrotra, S Sookhoo, S Kolla, H Halbert, K Lavell, S England

**Affiliations:** Department of Radiology, Sunderland Royal HospitalUnited Kingdom

**Keywords:** Computed tomography, Lumbar puncture

## Abstract

**Background and Aim**: In patients suspected of having a subarachnoid haemorrhage (SAH), a normal CT should be followed by lumbar puncture (LP) to detect xanthochromia. We studied the practice of performing a LP following a normal CT in patients with a clinical suspicion of SAH in a District General Hospital. We aimed to assess whether patients were being fully investigated for SAH and whether standards were being met.

**Methods**: This was a prospective study aiming to improve the patient's care by implementing the best practice. We initially recorded CT and LP results of patients with suspected SAH (phase 1) and presented the results to the referring clinicians. After a period of time, data was re–collected to study any change in practice (phase 2).

**Results**: In phase 1, 36 of 61 patients (59.0%) with a normal CT had a subsequent LP compared to 67/104 (64.4%) in the second phase (p=0.51). In the first phase, xanthochromia was detected in 1 of 36 patients (2.8%) who had a LP following a normal CT, compared to 1 of 67 patients (1.5%) in the second phase (p=1.0).

**Conclusion**: Approximately a third of patients with symptoms of SAH in both study periods did not undergo LP following a normal CT scan. This is an important finding, as it is known that a normal CT does not exclude the diagnosis of SAH and by not proceeding to LP, patients have not been fully investigated for a SAH.

## Introduction

Computed Tomography (CT) is the accepted first line investigation for patients with a suspected subarachnoid haemorrhage (SAH). SAH is associated with a normal CT in up to 2#x0025; of cases within 12 hours of onset of symptoms,[1] 6.9% within 24 hours[2] and 16.2% after 24 hours.[2] In order to identify this sub–group of patients, a lumbar puncture (LP) is mandatory following a normal CT to provide a cerebrospinal fluid (CSF) sample for bilirubin spectrophotometry. If present, CSF bilirubin will only be detected more than 12 hours after the onset of symptoms.[3] MRI imaging with FLAIR (fluid attenuated inversion recovery) techniques can demonstrate SAH as reliably as CT in the acute phase,[4] however, the MRI service provision in most District General Hospitals is limited. In addition, unwell patients may require aneaesthesia prior to a MRI examination.

We studied the practice of performing a LP following a normal CT in patients with a clinical suspicion of SAH in our unit. All of these patients had presenting symptoms of acute severe headaches with or without neurological deficit. Following the initial results, the clinicians were informed, so that any necessary changes could be implemented prior to the second phase of the study. We aimed to assess whether patients were being fully investigated for SAH and whether standards were being met.

## Materials and methods

All patients with sudden onset, severe headaches, suspicious of SAH who were referred for a CT scan were included in this study. Patients were clinically assessed prior to the CT scan by either a Registrar or Consultant in Medicine or Accident and Emergency. These patients were identified by the radiographers and details entered into a logbook housed in the CT department. Patients were scanned on Toshiba Asteion multi and single CT scanners. 

The initial data collection period was 23/1/2000 – 20/11/2002 (phase 1) and the results of this phase were presented to the referring clinicians. Following this, the second collection period was undertaken from 1/1/2003 to 29/11/2005 (phase 2) to study any change in practice. 

The time of admission and the date, time and CT report were obtained from the computerized patient information system. The CT results were divided into three categories: those in which features of SAH were present, completely normal CTs, and finally, CTs in which another pathology was identified, which could account for the patients’ symptoms (e.g. brain tumor). In addition, any mention in the CT report of whether a LP was recommended was also noted. Details of any subsequent LP, including the date, time and LP result were recorded from the computerized pathology information system. Statistical differences were identified with the Fishers exact test. 

## Results

**Figure 1:**
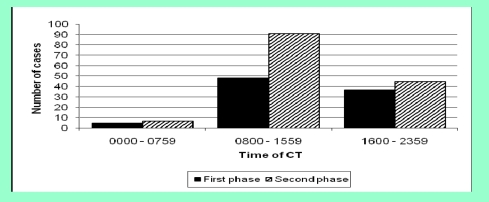
The time the CT was performed in patients with a clinical suspicion of SAH

There were 101 patients (41 males, 60 females) in the first phase and 143 patients in the second phase (58 males, 85 females). The age distribution in the first phase was 16–82 years old (median 44 years old) and in the second phase 18–90 years old (median 42 years old). In both study periods, more CTs were performed between July and September and the majority of CTs were performed in normal working hours, between 08:00 and 15:59 (Figure 1). In the first phase, 11 CT reports were not available; therefore, these patients were excluded from subsequent analysis.

### CT diagnosis

**Figure 2:**
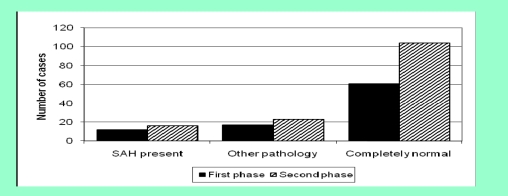
Diagnosis on CT report for patients suspected of having a SAH

In the first phase, 61/90 (67.8%) patients had a completely normal CT scan, compared to 104/143 (72.7%) in the second phase (Figure 2, p=0.460, not significant). 

In the first phase, thirty–seven (60.7%) of these patients had a CT within 12 hours of admission, 17 patients (27.9%) between 12 and 24 hours of admission and 6 patients (9.8%) were scanned more than 24 hours after admission (time of admission not available for one patient). In comparison, 67 patients (64.4%) in the second phase had a CT within 12 hours of admission, 25 patients (24.0%) between 12 and 24 hours and 12 patients (11.5%) more than 24 hours after admission, Other pathology noted on the CT scans in patients who did not have SAH included cerebral infarct (first phase: 7 cases, second phase: 8 cases), meningioma (1 case in each phase) and intracranial haemorrhage (first phase: 2 cases, second phase: 1 case).

### LP following normal CT scan

In the first phase, 36 of 61 patients (59.0%) with a normal CT had a subsequent LP compared to 67/104 (64.4%) in the second phase (p=0.509, not significant). In addition, in the first phase, 10/12 patients (83.3%) with a normal CT had a LP following recommendation in the CT report compared to 24/33 (72.7%) patients in the second phase. Furthermore, in the first phase, 7/17 patients (41.2%) with a normal CT which was performed 12–24 hours following admission didn't undergo a subsequent LP, compared to 14/25 patients (56.0%) in the second phase.  In the first phase of the study, 5/6 patients (83.3%) with a normal CT performed more than 24 hours following admission didn't undergo a subsequent LP, compared to 8/12 patients (66.7%) in the second phase.

**Figure 3:**
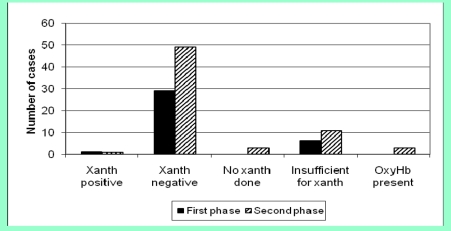
LP outcome following normal CT scan / (Xanth=xanthochromia, OxyHb=Oxyhaemoglobin)

In the first phase, xanthochromia was detected in one of 36 patients (2.8%) who had a LP following a normal CT compared to one of 67 patients (1.5%) in the second phase (Figure 3, p=1.000).

These patients were referred to the regional Neurosciences centre for angiography; the patient in the first phase had an anterior communicating artery aneurysm, which was coiled, the patient in the second phase did not have a vascular abnormality on angiography. In the first phase, six CSF samples were insufficient for xanthochromia analysis following a normal CT and only one of these was subsequently repeated (16.7%). In the second group, 11 samples were insufficient for xanthochromia analysis and, in three other cases xanthochromia was not analyzed. Two of these 14 samples were repeated (14.3%, p=1.000). 

**Figure 4:**
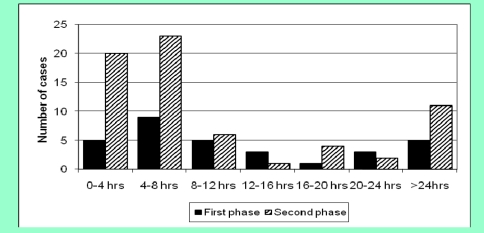
Timing of LP following normal CT scan / (hrs=hours)

The timing of LP following a normal CT scan is demonstrated in (Figure 4).

In the first phase, LPs were performed at various times following the normal CTH, whereas in the second phase the majority of LPs were performed within 8 hours of a normal CTH, although there was a second peak after 24 hours. Four patients in the first phase had a LP prior to the CT scan and the timing of the LP for a further patient was not known, therefore the data for these patients in not shown in (Figure 4).

## Commentaries of results

In the first phase of the study, 59.0% of the patients who had a normal CT subsequently underwent a lumbar puncture. Medical and Accident and Emergency Consultants who routinely manage patients with a suspected SAH were informed of these results at our hospital's clinical management meeting prior to the second phase of the study. At the time, it was suggested that more patients should undergo a LP following a normal CT scan if the clinical suspicion of SAH remains. Although, a greater proportion of patients with a normal CTH had a LP in the second phase (64.4%) compared to the first phase (59.0%), the difference does not achieve statistical significance. Therefore, the changes that we suggested following the results of the first phase have not been fully implemented.  

Overall, more patients in the present study underwent LP following a normal CT than previously reported by Perry et al (21.6%) and O'Neill et al (49%).[5,6] However, a large proportion of patients in both phases of the study with a normal CT performed more than 24 hours after admission, did not undergo subsequent LP. A delay in performing CT is associated with a decrease in the sensitivity for SAH and therefore these patients should all undergo LP for definite diagnosis.[1,2] Not all patients with symptoms suspicious of SAH in the present study have been fully investigated, with both CT and LP. One of the possible reasons for this may be that many patients with severe, non–traumatic headaches, which are not due to SAH, improve with analgesia and anti–emetics during hospital admission.[7] These patients may have initially presented with symptoms suspicious of SAH necessitating investigation with a CT scan. However, in the time following the CT scan, their symptoms may have improved and the clinical suspicion of SAH reduced. In addition, post–dural puncture headache (PDPH) is a significant clinical problem and has been reported in approximately a third of post lumbar puncture patients.[8] Therefore, in patients whose headache symptoms improved, a clinical decision may have been made to avoid a LP. This cannot wholly explain the fact that approximately a third of patients with a normal CT in both study periods did not undergo a subsequent LP. None of these patients subsequently returned to our hospital with a SAH diagnosed on CT or LP. However, SAH is often preceded by a warning headache and, to our knowledge, none of the patients with normal CT findings and no LP subsequently died in the community from SAH. However, following discharge from hospital, it is not known if any patients attended another hospital outside the region, with a SAH.[9] Although it is not always necessary to investigate all patients with a headache, the benefit of correctly identifying SAH is high, and, the results of this study highlight that many patients with clinical suspicion of SAH, are only being partially investigated by CT without subsequent LP. Clinicians are frequently reluctant to perform a LP following a normal CT partly due to the low yield of detecting SAH in this group and partly due to concern that LP frequently did not influence patient management.[10] This is also a likely causative factor for failure to perform LP after normal CT in the present study.

Another point that was highlighted at the clinical management meeting following the first study period was that LPs were performed at various intervals following a normal CT scan. In particular, five LPs were performed more than 24 hours after the normal CT scan. Although xanthochromia will be detected between 12 hours and 2 weeks in CSF after the onset of a bleed from a SAH, it is still necessary to perform a LP at the earliest possibility, so that patients subsequently diagnosed with SAH can receive the correct management. This is particularly important as there is a significant risk of mortality due to re–bleeding following SAH [11] and patients with warning bleeds subsequently have worse outcomes compared to those without warning bleeds.[12] Although the time of onset of ictus was not known in this study, we have analyzed the investigation pathway for SAH in our unit and we highlighted the areas which can be further improved. In the second study period, 11 patients (16.4%) had a LP more than 24 hours after CT; therefore, the changes suggested at the clinical management meeting have not been implemented. One possible reason for this is that many patients in both study periods had a CT in the evening time. The junior doctors managing the patient at this time may not have been aware of the need to further investigate the patient with a LP.

There has also been no significant change in patients having a repeated LP following an initial one, insufficient LPs between the two study periods. The possible reasons for an insufficient CSF sample include operator inexperience in performing an LP, a technically difficult LP and also the operator being unaware of the minimum quantity of CSF required for xanthochromia analysis. The possible reasons for not repeating a LP after an insufficient CSF sample may be patient in–cooperation or improvement of symptoms. However, the same risk of failing to diagnose a SAH remains.

Radiologists may suggest the next step in the investigation armamentarium depending on the findings and the clinical information given. This can be particularly helpful with the present shortened length of medical attachments for junior doctors which may result in doctors being inexperienced in a particular specialty.  This study presents a valuable insight into the investigation pathway of SAH in a typical District General Hospital.

## Conclusion

In summary, approximately a third of the patients with symptoms of SAH in both study periods did not undergo a LP following a normal CT scan. This is an important finding as it is known that a normal CT scan does not exclude the diagnosis of SAH and by not proceeding to LP, patients have not been fully investigated for a SAH.[1,2] A combination of junior doctor education and formalizing the investigation pathway for patients suspected of having a SAH may be necessary.
